# Establishing an MSU service in a medium-sized German urban area—clinical and economic considerations

**DOI:** 10.3389/fneur.2024.1358145

**Published:** 2024-02-29

**Authors:** Johann S. Rink, Fabian Tollens, Andrej Tschalzev, Christian Bartelt, Armin Heinzl, Jens Hoffmann, Stefan O. Schoenberg, Annika Marzina, Vesile Sandikci, Carla Wiegand, Carolin Hoyer, Kristina Szabo

**Affiliations:** ^1^Department of Radiology and Nuclear Medicine, Medical Faculty Mannheim, Mannheim University Medical Centre, Heidelberg University, Mannheim, Germany; ^2^Institute for Enterprise Systems, University of Mannheim, Mannheim, Germany; ^3^University of Mannheim, Mannheim, Germany; ^4^Department of Neurology, Medical Faculty Mannheim, Mannheim University Medical Centre, Heidelberg University, Mannheim, Germany

**Keywords:** acute stroke care, mobile stroke units, prehospital stroke care, computerized tomography, prehospital thrombolysis

## Abstract

**Background and purpose:**

Mobile stroke units (MSU) have been demonstrated to improve prehospital stroke care in metropolitan and rural regions. Due to geographical, social and structural idiosyncrasies of the German city of Mannheim, concepts of established MSU services are not directly applicable to the Mannheim initiative. The aim of the present analysis was to identify major determinants that need to be considered when initially setting up a local MSU service.

**Methods:**

Local stroke statistics from 2015 to 2021 were analyzed and circadian distribution of strokes and local incidence rates were calculated. MSU patient numbers and total program costs were estimated for varying operating modes, daytime coverage models, staffing configurations which included several resource sharing models with the hospital. Additional case-number simulations for expanded catchment areas were performed.

**Results:**

Median time of symptom onset of ischemic stroke patients was 1:00 p.m. 54.3% of all stroke patients were admitted during a 10-h time window on weekdays. Assuming that MSU is able to reach 53% of stroke patients, the average expected number of ischemic stroke patients admitted to MSU would be 0.64 in a 10-h shift each day, which could potentially be increased by expanding the MSU catchment area. Total estimated MSU costs amounted to € 815,087 *per annum*. Teleneurological assessment reduced overall costs by 11.7%.

**Conclusion:**

This analysis provides a framework of determinants and considerations to be addressed during the design process of a novel MSU program in order to balance stroke care improvements with the sustainable use of scarce resources.

## 1 Introduction

Current guidelines of the European Stroke Organization recommend the use of Mobile Stroke Units (MSUs) over conventional care for the prehospital management of patients with suspected stroke (1). First introduced in Germany in 2003 (2), MSUs are specialized ambulances equipped with stroke care teams, CT scanners and point-of-care laboratory testing devices, which allow for the prehospital diagnosis and treatment of acute stroke. In recent years, MSU utilization has been demonstrated to positively impact various dimensions of stroke care such as the speed and frequency of intravenous thrombolysis (IVT), and stroke outcome (3–5). So far, MSU services have been confined to research settings in either large metropolitan areas or sparsely populated rural regions and have been highly individualized according to local conditions, regulations and restrictions. Economic factors are not negligible since costs of MSU programs, which range from approximately € 550,000 to 1.8 Million annually (4), are not regularly reimbursed by health insurance funds.

Mannheim is the second-largest city in the German state of Baden-Wuerttemberg, with a population of more than 310,000 inhabitants and a surface area of 145 square kilometers. A current government-funded project affords the unique opportunity to establish a local MSU service. Mannheim’s geographical, social, healthcare-and traffic-related infrastructural properties, however, differ substantially from those of regions with already established MSU services, necessitating a concept tailored to site-specific idiosyncrasies. This paper outlines critical steps in this process in conjunction with economic modeling against the backdrop of the present status of local stroke care.

## 2 Materials and methods

### 2.1 Analysis of local stroke statistics

Stroke incidence in Mannheim and hence prospective patient numbers were estimated based on data available from the two local stroke units (6, 7). Furthermore, we analyzed data of all patients with the diagnosis of acute ischemic stroke (AIS), who received IVT and/or mechanical thrombectomy (MT) and were admitted to the Comprehensive Stroke Center, Department Neurology, University Hospital Mannheim, Germany between 01 December 2015 and 31 December 2021. We extracted data concerning the time of onset and arrival at hospital. We analyzed the distribution of patient arrival frequency over the course of a 24-h period. This study adheres to the observational cohort guideline, documented via the STROBE checklist (8).

### 2.2 General assumptions for the prospective MSU service in Mannheim

The MSU will be stationed at the Mannheim University Medical Center, a central position relative to Mannheim’s limits of landmark and of excellent accessibility. The initial catchment area is represented by the city borders of Mannheim, which corresponds to driving distances of approximately 15 min under emergency conditions. The total operating time of the MSU service is estimated at 292 days (200 workdays) per year, considering time required for MSU maintenance and staff training. Different modes of operation were defined based on practical considerations: 8-, 10-, 16-, or 24-h daily operation was simulated for 3, 4, 5, 6, and 7 days per week. The basic staffing model consists of a team of three healthcare professionals: one neurologist, one radiologist technician, and one paramedic. The MSU will be deployed upon dispatch whenever possible and a conventional emergency medical service (EMS) vehicle will be dispatched alongside the MSU. To maximize availability for on-site stroke management, the MSU is not designed for patient transportation to the hospital. However, for post-thrombolysis patient transfer to the hospital, the MSU neurologist may join the regular EMS crew in a case-dependent decision, depending on the required level of care.

The estimated proportions of patients with ischemic stroke, transient ischemic attack (TIA), hemorrhagic stroke, and stroke mimics typically treated by MSU were taken from recent literature (9, 10). MSU-specific metrics such as the number of cancelations, utilization rate, average distance and duration per dispatch, as well as the tPA administration rate were considered (11, 12). Assuming an MSU can miss stroke cases on a dispatch-level by inaccuracy of EMS dispatchers and on an MSU-level by not being able to manage simultaneous cases at peak times and by being located too far away from a specific case. Given the reported very heterogeneous rates of 22.3%–51.1% dispatcher sensitivity for stroke (13–16), MSU can be expected to partly compensate for this inaccuracy by strategies like attending non-stroke code dispatches. With a limited total number of ischemic stroke cases in 10 h per day, and most cases happening around noon, the number of simultaneous cases for the Mannheim scenario can be expected to be limited. Also, due to the compact catchment zone, there will not be many cases in which MSU will be too far away from a case. Therefore, a total combined number of 50% missed cases were chosen for modeling. All parameters included in the model are presented in Supplementary Table S1.

### 2.3 Patient numbers, coverage, and cost analysis

The number and proportion of ischemic stroke patients admitted to the Comprehensive Stroke Center was evaluated for different operating times (Table 1). Absolute numbers of expected ischemic stroke cases for the MSU were estimated by proportional adaptation with published case statistics from the second stroke unit in Mannheim, combined with further reduction of MSU-missed strokes cases. Percentage treated with IVT was estimated based on hospital records. Openrouteservice online service (17) was used to estimate the covered population and MSU-admitted cases for potentially larger catchment zones according to driving time around the MSU base station. 30% of reduced driving time under emergency conditions was hypothesized, based on experience from the local dispatch center.

**Table 1:** Proportion of ischemic stroke patients admitted to the hospital in 8- (9 a.m.–5.p.m.), 10- (9 a.m.–7 p.m.), 16- (8 a.m.–12 p.m.), and 24-h time windows from Monday to Wednesday (3 days), Monday–Thursday (4 days), Monday–Friday (5 days), Monday–Saturday (6 days) or Monday–Sunday (7 days), and possibly accessible by MSU.

	3 days	4 days	5 days	6 days	7 days
8 h operation	28.75%	37.91%	46.77%	54.29%	60.36%
10 h operation	33.01%	43.77%	54.33%	63.07%	70.21%
16 h operation	41.87%	55.85%	68.85%	80.12%	90.63%
24 h operation	45.59%	61.42%	75.90%	87.97%	100.00%

Cost estimation was based on costs of initial investments and running costs attributable to hardware, management and staffing costs (Table 2). In order to create realistically replicable cost projections independent of specific vendors, the cost of suitable components for MSU was estimated based on local sources, particularly hospital accounting and subunits as well as estimations of EMS and the regional rescue dispatch and control center. Market prices for exemplary components or services were used whenever available. All cost data were deflated to 2022 Euros using the German consumer price index (Table 2; Supplementary Table S1). Initial investment costs were depreciated over 6 years. Recurring expenses were considered for the medical hub, the dispatch and control center as well as the EMS. Labor costs included personnel costs for the MSU team, its administration and its training. Fuel consumption was captured according to MSU-run average distance and frequency. Costs for telemedicine consultations and costs for increased t-PA use by MSU were considered, and savings by avoiding intrahospital CT-scans were taken into account. For a 5-days-per-week simulation, average MSU costs per ischemic stroke patient were calculated for the different operating modes.

**Table 2:** Cost estimation based on hospital accounting, local EMS, dispatch center, and literature.

Parameter	Value
**Aggregated hardware and project set-up costs (before taxes)**	
Total hardware investments including vehicle platform, customization, CT scanner and telemedicine equipment	€ 856,953
Total medical equipment	€ 34,889
Permissions / licenses	€ 3,127
Initial investments at dispatch and medical hub	€ 111,000
Total (before taxes)	€ 1,005,968
**Aggregated running costs, per annum (before taxes)**	
Total MSU hardware maintenance	€ 52,958
Total medical supplies	€ 2,310
Insurance	€ 12,480
Running costs at medical hub, EMS and dispatch (infrastructure, training)	€ 75,344
Total (before taxes)	€ 131,666
**Aggregated staffing costs (including taxes)**	
Administration overhead for personnel costs	Additional 22% of costs
Emergency paramedic	€ 74,997
Radiology technician	€ 63,547
Radiology/Neurology resident	€ 92,045
Attending physician (Neurology and Radiology)	€ 135,996
Project management	50% of attending physician

Where unavailable, costs were estimated based on expert interviews. Detailed information on costs and calculations are provided in the Supplementary material.

Three alternative staffing scenarios were simulated: the main scenario consisted of an MSU team including full-time positions of a neurologist, a radiologist technician, and a paramedic, as this is the anticipated initial set-up. The hospital radiologist on call covers the unit remotely on all scenarios. As the Mannheim MSU can return to the hospital in between dispatches, a second alternative scenario is possible in which the neurologist only joins the MSU team for each dispatch and – according to a resource sharing agreement with the hospital—only this fraction of physician staffing costs are being reimbursed from the MSU project budget. In the third scenario, the neurologist assesses the patients remotely and its personnel costs are being reimbursed by the MSU project only for the time spent with telemedicine consultations. Costs of teleneurological and teleradiological assessment were estimated based on hourly wages and average duration of consultations (18).

## 3 Results

### 3.1 Local stroke care

**Figure 1:**
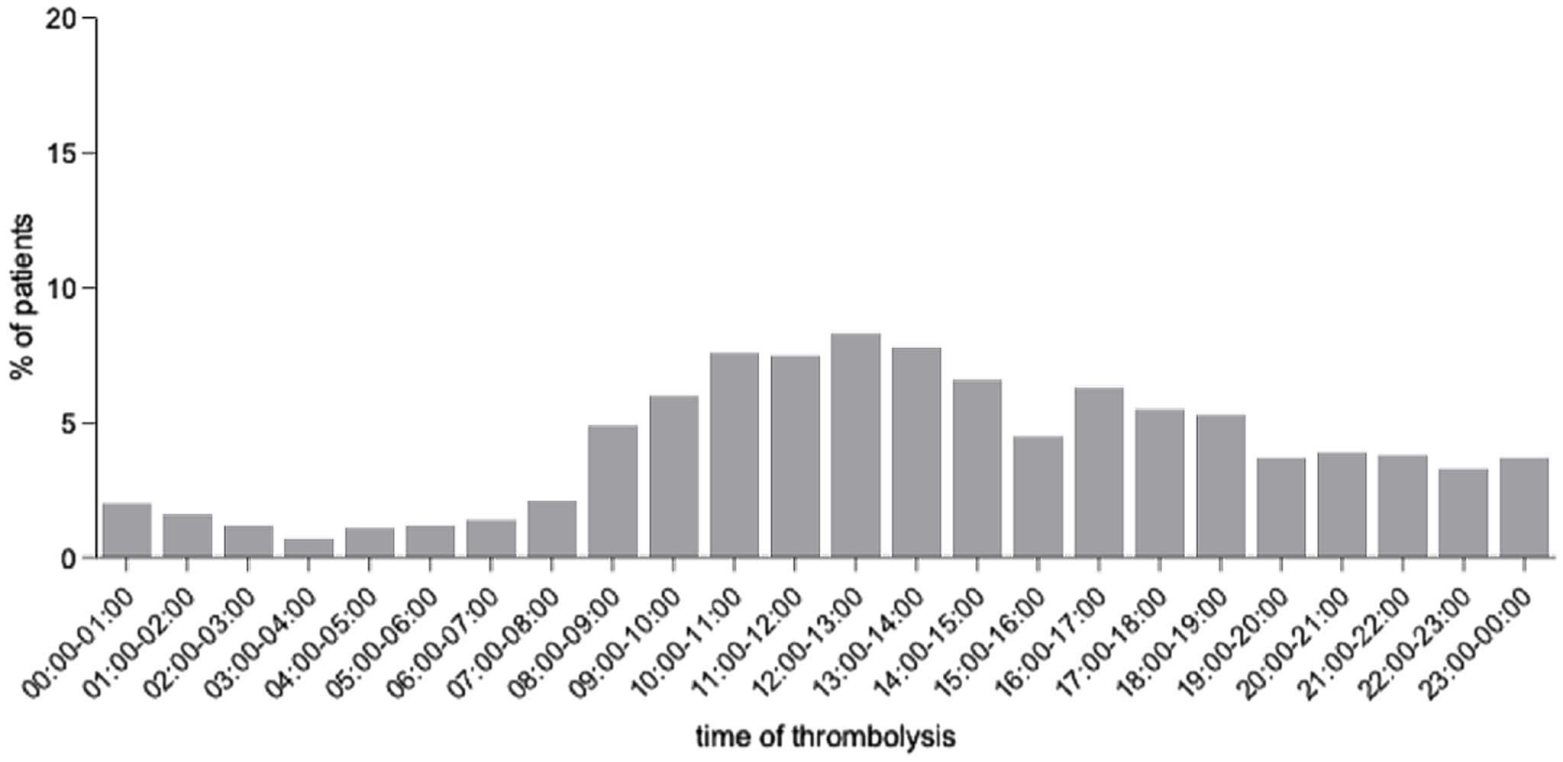
Analysis of average time of day of thrombolysis initiation in a series of 1,775 patients receiving intravenous thrombolysis and a subset of 245 patients treated with mechanical thrombectomy.

During the analyzed period, a total of 5,221 patients with AIS were treated in our stroke unit; 1,175 patients (22.5%) received IVT and MT was performed in 610 cases (11.6%). For 5,187 patients, time of hospital admission was available and for 837 of the thrombolyzed patients. Median time of symptom onset was 1:00 p.m. (Q1: 9:52 a.m.; Q3: 5:00 p.m.), median time of hospital admission was 1:57 p.m. (Q1: 11:04 a.m.; Q3: 5:59 p.m.). The majority of patients received IVT between 8:00 a.m. and 7:00 p.m., while IVT rates were lower outside of this time range (Figure 1).

### 3.2 Operation modes, case coverage and local incidence

During the investigated period, there were 642 days (29.3%) without any ischemic stroke admissions to the University Hospital and fewer stroke patients were admitted on weekends than during weekdays (1.034 vs. 1.316 per day on average, *p* ≤ 0.001).

Considering the average circadian distribution of stroke admissions over the 6 years, 59.5% of thrombolyzed patients were admitted within an 8-h time window (9 a.m. to 5 p.m.), whereas 65.4% and 88.7% of patients were admitted in a 10-h (9 a.m.–7 p.m.) and 16-h (8 a.m.–midnight) time window, respectively (Table 1). Based on stroke statistics of the two stroke units in Mannheim, 74.8% of reported strokes were treated by the Comprehensive Stroke Center and 25.2% of cases by the second stroke unit. The resulting local stroke incidence in the catchment area was estimated at 184.48/100,000 inhabitants per year.

**Figure 2:**
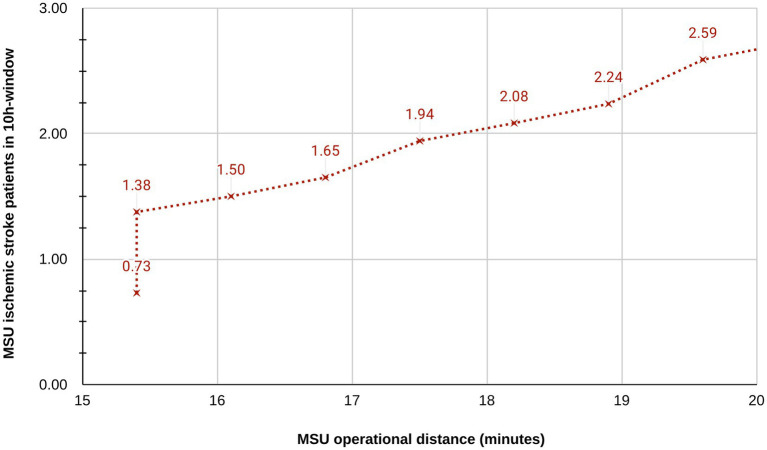
Simulated expansion of catchment zone: Association between driving distance around MSU base station and simulated MSU patient numbers. The sharp increase between 15 and 16 min occurs when surrounding areas outside the city borders are integrated into the catchment area.

Considering missed cases of 50% (additionally 60%–40%), expected numbers of MSU-treated patients according to days of MSU operation per week and daily hours of operation along with number of IVT treatments are shown in Table 3. For a 10-h day, 0.64 (0.51–0.76) ischemic stroke patients would be admitted to the MSU, leading to an average of 0.13 (0.11–0.16) IVT treatments per day. The expected number of additional MSU-managed TIA, ICH and mimics patients is shown in Supplementary Table S3. Simulated expansion of MSU driving distance around the base led to an increase of expected patient numbers, as displayed in Figure 2.

**Table 3:** Modeled ischemic stroke patient numbers and numbers of t-PA treatments in MSU per weekday, according to the operational model, assuming the rate of 50% missed strokes.

	Average number of ischemic stroke patients/day	Average IVT treatments/day	Average MSU costs per ischemic stroke patient
8 h operation	0.55 (0.44–0.67)	0.12 (0.09–0.14)	€ 7,809
10 h operation	0.64 (0.51–0.76)	0.13 (0.11–0.16)	€ 7,907
16 h operation	0.77 (0.61–0.92)	0.16 (0.13–0.19)	€ 7,739
24 h operation	0.84 (0.68–1.01)	0.18 (0.14–0.21)	€ 9,273

The last column shows average MSU costs per ischemic stroke patient.

### 3.3 Cost analysis

For the standard model with a 10-h shift on 5 days per week, total annual costs amounted to € 815,087 (Supplementary Figure S1). Personnel costs including project management and training time accounted for the major component (58.0% of budget), whereas annual expenses for initial investment (23.9%) and running costs (22.6%) were smaller.

**Figure 3:**
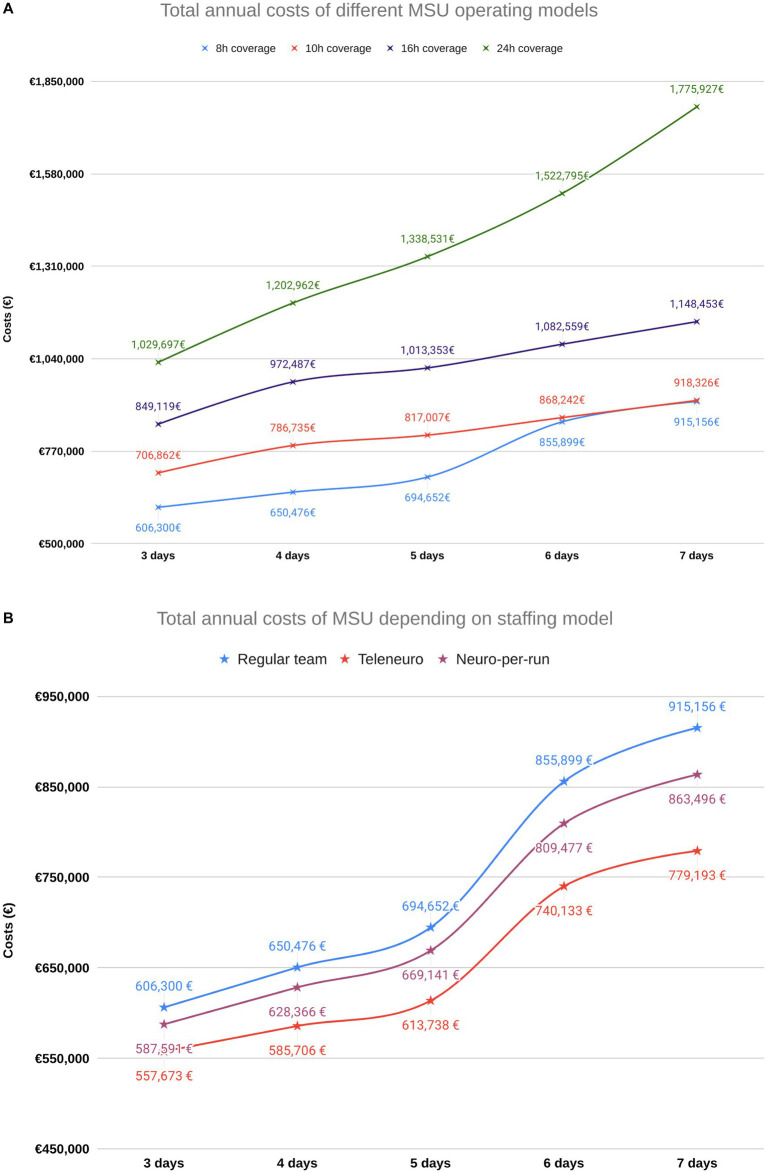
Cost simulations for various operating modes. (A) Overall program costs vary substantially depending on operating times. (B) For an 8-hour per day operation, the different possible staffing configurations lead to different resulting program costs.

Overall costs varied depending on operating times and staffing (Figure 3). Reducing MSU staffing either by excluding the neurologist from MSU between cases or full removal from the vehicle by telemedicine resulted in an overall cost reduction of 3.7% and 11.7%, respectively.

## 4 Discussion

Multiple MSU projects in Europe and the United States have demonstrated the potential to reduce time to IVT and to improve functional outcomes in ischemic stroke patients (19). This study evaluated various operating modes, based on clinical and financial data from our institution, with the aim of conceptualizing a feasible way to initiate an MSU service tailored to the conditions and needs of a medium-sized German city.

A paramount objective for MSUs is to shorten the latency between stroke recognition and therapy initiation, thus effectively increasing overall IVT rates and in particular, rates of ‘golden hour’ thrombolysis, i.e., intravenous thrombolysis within the first 60 min after symptom onset. IVT rates in Germany average at 16.3% with a substantial degree of regional variation (20). An IVT rate of over 20% in the city of Mannheim indicates an already well-established system of conventional local stroke care, and it remains to be seen if a further increase of the IVT rate, especially a leftward shift of the onset-to-needle time distribution, can be accomplished. With a central base of deployment, it can be expected that average alarm-to-on-site arrivals will take longer for the MSU than for conventional EMS vehicles, which are deployed depending on their current location and the shortest distance to the scene. However, due to advancing therapy initiation, it is likely possible for MSUs to compensate for these potential delays and achieve shorter onset-to-treatment times as a far more critical medical performance indicator. This would also hold for patients eligible for MT as those could be identified on-site and transported directly to the angiography suite of our comprehensive stroke center.

In this study, the city of Mannheim represented the initial catchment zone since driving times of approximately 15 min matched with the borders of the city, mainly defined by structure of local EMS and dispatch center. Meaningful expansion of the catchment zone is an anticipated goal of the local effort. In this regard, a rendezvous model could be considered to further increase the catchment area.

MSU programs worldwide have reported highly individualized operating times and modes (21). Assuming 10 operating hours per day Monday to Friday, 54.3% of all weekly strokes would potentially be accessible for MSU. To achieve a coverage of >75%, 16 h operation including weekends would be necessary.

Including realistic rates of missed strokes is essential. In general, while prehospital stroke scales for detection of stroke as well as estimation of stroke severity show acceptable sensitivity and specificity (22), dispatcher accuracy of stroke recognition is modest with considerable variation (13–16, 23). Optimizing this challenging part of the rescue chain is a major area of interest in prehospital stroke management (24), which directly pertains to the operation of MSU services. Patients with stroke mimics such as seizures and non-ischemic strokes, for example intracranial hemorrhage, also benefit from early neurologic assessment and imaging (25, 26).

Expected average daily patients treated by MSU ranged from 0.55 (0.44–0.67) to 0.77 (0.61–0.92) in 8- to 16-h operation models. The rate of daily t-PA administration per MSU is expected to be 0.22 (0.18–0.26) per 24 h, which is nearly five-fold lower than the one reported by the well-established MSU service from Houston, TX (12). Whereas MSU programs in rural areas aim to expand the radius of MSU operation up to 250 km (27), in the Mannheim case with a densely populated surrounding area, simulation revealed that a limited increase in driving distance of five more minutes lead to an increase of 30–40% in patient numbers. Evidence from the projects in Berlin and Houston which reported covered populations of 1.3 Million (28) and 2 Million (29) inhabitants and operate in part in rendezvous mode, further underline the ability of MSUs to serve larger communities. The main challenge, however, is to define areas in which MSUs add the highest value to the rescue chain and, in the Mannheim case, to efficiently integrate overlapping EMS systems and hospitals from three different federal states. From the perspective of healthcare policy design and resource allocation, accurate data on treated strokes, stroke incidence, and missed strokes are essential metrics for estimating the clinical and economic potential of MSUs for various settings. Consequently, dispatch criteria, rates of stroke mimics, local organizational characteristics of EMS and their impact on stroke coverage rates are areas worthy of more rigorous investigation.

Different operational models enable the MSU service to cover a specific proportion of stroke cases. Expected costs varied highly and ranged from approximately € 550,000 to € 1,900,000, respectively. Cost-analysis identified that staffing accounts for the largest fraction of costs, leading to a high dependence of total costs on the choice of operating times. This finding is in line with published analyses from two German MSU projects, the Norwegian and the Australian project (30–33). It underlines that, depending on availability of funding and personnel, there are different options to initiate and operate an MSU service. In our estimation, a cost reduction by 11.7% in the standard model was possible by replacing an on-board neurologist by teleneurological assessment, which was previously described as a clinically feasible option (34, 35). The option to join the MSU only for dispatches or to reduce costs by telemedicine however will only be possible, if there is a resource sharing agreement in place, which enables the MSU project to instantly use resources from neurologists and radiologists which are in general compensated by regular hospital contracts. Up to now, regulatory restrictions require radiology technicians and, in case of the use of contrast agents, also physicians to be on scene in Germany. Investment decisions on different hardware components resulted in a rather small impact on the annual project budget. Hence, meaningful hardware investment decisions should not be constrained by economic concerns alone. When looking at average MSU costs per ischemic stroke patient, the models of 8-, 10-, and 16-h daily operation relatively uniform yielded costs around € 7,500, whereas the 24-h operational model yielded higher costs of nearly € 9,000. From an economical perspective, daytime operation from 8 to 16 h appears most reasonable in view of scarce financial and staffing resources. Despite substantial resources needed for MSU operation, economic data from the Melbourne MSU project (32) and from both the German Homburg (30) and Berlin-based (31, 36) MSU services demonstrate that avoidance of disability bears the potential to render the public investment into MSUs cost-effective. An area of further future investigation will be the impact of the use of tenecteplase for ischemic stroke. The substance has not yet been approved for this indication in Germany but appears to positively influence cost-effectiveness (37).

This is the first analysis examining coverage and costs of different MSU program configurations in Germany. The highly uniform cost structure and tariffs in the German healthcare system allows for a straightforward translation of most of our findings to other sites while model variables such as catchment area populations need to be adjusted when considering MSU patient numbers. The present analysis is limited by several factors: model outcomes depend on estimated variables such as catchment area population, rates of stroke mimics and missed strokes. Local stroke incidence in the catchment zone was estimated based on hospital records and published data by the two local stroke units. These variables potentially influence stroke case numbers as well as overall program costs. Furthermore, this model did not include factors like daytime-dependent traffic delays and variations in missed cases according to daytime and catchment zone size. Cost estimates for hardware and services were mainly based on exemplary components that are commercially available; however, the costs of some components had to be estimated, causing some degree of inaccuracy. To address these issues, estimates were chosen conservatively.

In conclusion, our analysis demonstrates that circadian distribution of stroke admissions should be considered when planning operating modes of MSU projects. When choosing an operational model, local factors like availability of funding and personnel, but also organizational structures of local dispatch and EMS, need to be considered. Operation from at least 5 days per week and 8 to 16 h per day appears to be an appropriate strategy to initiate an MSU service in Mannheim, additionally, the use of telemedicine is favorable. To continuously improve organizational aspects of MSU programs and enable a transfer to different settings, further data on different operating modes, rates of stroke mimics, missed strokes, and catchment zone population details is required. Integrating these variables during MSU program set-up will allow for balancing the needs related to stroke care improvement with economically acceptable resource allocation.

## Data availability statement

The raw data supporting the conclusions of this article will be made available by the authors, without undue reservation.

## Ethics statement

The studies involving humans were approved by Ethik-Kommission II der Universität Heidelberg Medizinische Fakultät Mannheim. The studies were conducted in accordance with the local legislation and institutional requirements. The ethics committee/institutional review board waived the requirement of written informed consent for participation from the participants or the participants’ legal guardians/next of kin because the retrospective data acquisition has no influence on the treatment process.

## Author contributions

JR: Supervision, Validation, Writing – original draft, Writing – review & editing, Funding acquisition, Methodology. FT: Conceptualization, Data curation, Formal analysis, Investigation, Methodology, Validation, Writing – original draft, Writing – review & editing. AT: Data curation, Validation, Writing – review & editing. CB: Project administration, Resources, Writing – review & editing. AH: Funding acquisition, Resources, Writing – review & editing, Supervision. JH: Investigation, Visualization, Writing – review & editing. SS: Funding acquisition, Resources, Writing – review & editing, Supervision. AM: Conceptualization, Validation, Writing – review & editing. VS: Data curation, Visualization, Writing – review & editing, Investigation. CW: Validation, Writing – review & editing. CH: Investigation, Methodology, Supervision, Validation, Writing – review & editing. KS: Funding acquisition, Methodology, Project administration, Resources, Supervision, Validation, Writing – original draft, Writing – review & editing.
