# Supplementary material for: Establishing an MSU service in a medium-sized German urban area—clinical and economic considerations
Source: Front Neurol. 2024 Feb 29;15:1358145. doi: 10.3389/fneur.2024.1358145 (PMC10938346; doi:10.3389/fneur.2024.1358145)
Supplement: Supplementary file 1 [file Data_Sheet_1.docx]

Supplementary Material

# Table S1 – Cost model input parameters

Input parameters of the cost analysis. When no data were available, input parameters were based on literature, local EMS, hospital accounting, or expert estimates,

| **Parameter** | **Value** | **Source** |
| --- | --- | --- |
| Average daily total ischemic and hemorrhagic stroke patients from catchment zone presenting within 8h/10h/16h/24h time frames  Based on published case statistics by the second stroke unit in Mannheim, 25,2% of cases were added  Sub analyses for the different weekdays were calculated and used for modeling. | 1,308 / 1,498 / 1,804 / 1,992 | Hospital patient records and official published case statistics of the two local stroke units reporting the total number of complex neurological treatment of stroke patients ^1,2^ |
| Catchment zone population Mannheim | 306,982 | German Federal Office of Statistics (Statistisches Bundesamt), Average for 2015-2018 ^3^ |
| Extended catchment zone population, according to driving radius around MSU base in min, 30% faster | 15.4 min: 306,982 within city borders of Mannheim  15.4 min - 578,624  16.1min - 631,005  16.8 min - 694,249  17.5 min - 816,609  18.2 min - 876,475  18.9 min - 940,890  19.6 min - 1,089,108  20.3 min - 1,149,817  21.0 min - 1,232,589  21.7 min - 1,306,446  22.4 min - 1,387,936 | Openrouteservice web service ^4^, driving time 30% faster under emergency conditions, according to the local dispatch center. |
| **Patient / MSU metrics** | | |
| Proportion of intracranial hemorrhages from all strokes | 17% | Hospital patient records |
| Additional rate of transitory ischemic attacks (TIA) in relation to ischemic and hemorrhagic strokes | 63% | Based on reported rates from the German Erlangen stroke project ^5^ |
| Proportion of stroke mimics in MSU | 50% | Estimated upon Fassbender et al 2023 ^6^ |
| Fraction of missed strokes by MSU (dispatch-level / MSU-level) | 50% | Estimate based on Ellens et al 2022 ^7^, Mattila et al 2019 ^8^ Mould-Millman et al 2018 ^9^ Nour et al 2020 ^10^, Eliakundu et al 2022 ^11^ Bohm and Kurland 2018 ^12^ |
| Average driving distance (for estimation of fuel consumption) | 3,57 km | Calculation of average distance between CSC and confirmed stroke case dispatches from hospital data |
| Average total duration of one MSU run (to estimate staff resource use) | 65 min | Estimate, based on Ellens et al 2022 ^7^ |
| Length of typical teleconsultations between hospital and MSU | 4 min (Neurology) and 15 min (Radiology) | Weinberg et al ^13^ (Neurology) and own experience in acute stroke imaging (Radiology) |
| Additional runs per stroke (stroke mimics, TIA, en-route cancellations, logistic problems, etc.) | 3 | Estimate based on Ellens et al 2022 ^7^, Fassbender et al 2023 ^6^ and expert feedback from other projects |
| MSU operational time / year excluding training and holidays | 292 days / 200 weekdays | Project estimate * |
| Increase in t-PA administration rates in ischemic strokes / TIA / mimics by MSU | 12,20% | Calculated upon Ebinger et al 2021 ^14^ |
| **Hardware and project set-up costs (before taxes)** | | |
| Depreciation of all hardware / initial set-up investments | 6 years | Estimate by local EMS |
| Ambulance chassis carrier | € 70,000 | Project estimates * |
| Customization of vehicle for CT fixation and customization according to local requirements, shielding | € 160,000 |  |
| CT Scanner | € 489,082.86 | Reported costs by Dietrich et al 2014 ^15^ / Gyrd-Hansen et al 2015 ^16^ for the projects in Homburg and Berlin, doubled to reflect anticipated availability of new generations of CT scanners |
| Transmission hardware and software in ambulance / IT- and network set-up investment in medical hub | € 45,000 + € 25,000 + € 50,000 | Project estimates (mainly based on literature) * |
| Contrast agent injector | € 40,000 |  |
| Patient stretcher | € 24,300 | Estimate by local EMS |
| Costs medical equipment:  Monitor and Pulsoximeter /  blood pressure measurement equipment /  Coagulation measurement device /  Portable ultrasound device /  ECG + defibrillator /  Backpack /  Perfusors for t-PA application /  Oxygen bottle rent + valve + application equipment /  CPR board | € 4,281.00  € 40.00  € 880.00  € 4,289.00  € 19,440.00  € 810.00  € 3,577.50  € 384.58  € 124.74 | Project estimate * (mainly based on hospital accounting and local EMS data) |
| Lead protective gear, 2x | € 1,053.00 | Online source |
| Permissions and licenses (CT operation + teleradiology / ambulance registration) | € 2,600 / € 526.50 | Project estimate * (Estimate by radiation protection department, literature and local EMS) |
| Initial dispatch training and implementation / EMS training | € 35,000 / 26,000 |  |
| **Running costs (yearly, before taxes)** | | |
| Technical maintenance vehicle /  medical equipment /  CT scanner / | € 4,050.00 /  € 502.00  € 48,908.29 / | Local EMS (Ambulance, medical equipment),  for the CT scanner 10% of estimated initial costs were assumed |
| Medical supplies | € 2,310 | Local EMS, Hospital accounting |
| Insurance / licenses for the vehicle | € 12,480 | Local EMS |
| Continuous training, workflow implementation, data aggregation at Dispatch / EMS | € 12,000 / € 15,000 | Project estimate * |
| Parking lot including electricity costs | € 7,921.21 | Local EMS |
| Medical hub: IT Infrastructure, Mobile Data Service, Transmission software licenses and -maintenance contracts | € 40,000 | Project estimate * |
| **Case-dependent costs** | | |
| Fuel / km | € 0.15 | Local gas station, considering typical mileage of ambulance |
| Emergency department savings for avoidance of head-CT | € 108.72 | EBM 34310 + 34343 ^17^ |
| Cost of Actilyse® (70mg average) | € 904,29 | Local pharmacy |
| **Staffing costs (including taxes and all benefits)** | | |
| Administration overhead for personnel costs | Additional 22% of costs | Established research funding standards ^18^ |
| Emergency paramedics of the categories “Rettungsassistent” and “Rettungssanitäter” | € 74,997 | Local EMS |
| Radiology tech | € 63,546.96 | Hospital Accounting |
| Radiology / Neurology resident MSU | € 92,044.80 | Hospital Accounting |
| Attending physician (Neurology and Radiology) | € 135,996 | Hospital Accounting |
| Project management | 50% of attending physician | Project estimate * |
| Extra benefits for shift changes (monthly) / night hours / saturday afternoon / sunday / holiday | € 40 / +20% / +20% / +25% / +35% | Excerpt of the official wage agreements ^19^ |
| Annual external emergency training for physician / radiology tech | € 1,179 /  € 1,499 | Commercially available training courses ^20^ |

* Estimates were made by the authors. The numbers reported here have been thoroughly researched and validated by the authors in cooperation with multiple subunits of the hospital, hospital accounting, local EMS. They represent estimates which seem realistically replicable throughout Germany, independent from specific products by specific vendors as there are multiple possibilities combining hardware, software and third-party services in an MSU. In many cases, average costs for different possible products were calculated. For the CT scanner, substantial reserves were factored in as new generations of equipment from multiple vendors are estimated to become available. No direct cost-estimates for specific products were included.

# Costs of personnel

Staffing costs consisted of personnel costs for MSU team, administration and training. Unit costs were drawn from nationwide collective agreements and local hospital accounting, additional costs for night or weekend shifts were added according to these agreements. Project management was estimated to be provided by a physician in 50% part-time. Up-front training costs for neurologist and radiology technician were estimated based on available training courses, another month of paid training was added to each team member annually and accordingly subtracted from availability for MSU. An overhead of 22% extra costs for each person on the team was added.

# Costs breakdown

**Figure S1:** Breakdown of total program costs in a 10h/day-5 days coverage model. Personnel and training costs account for 58.0% of costs, whereas hardware and set-up investments account for 23.9% of costs, and yearly running costs account for 22.6%.

**
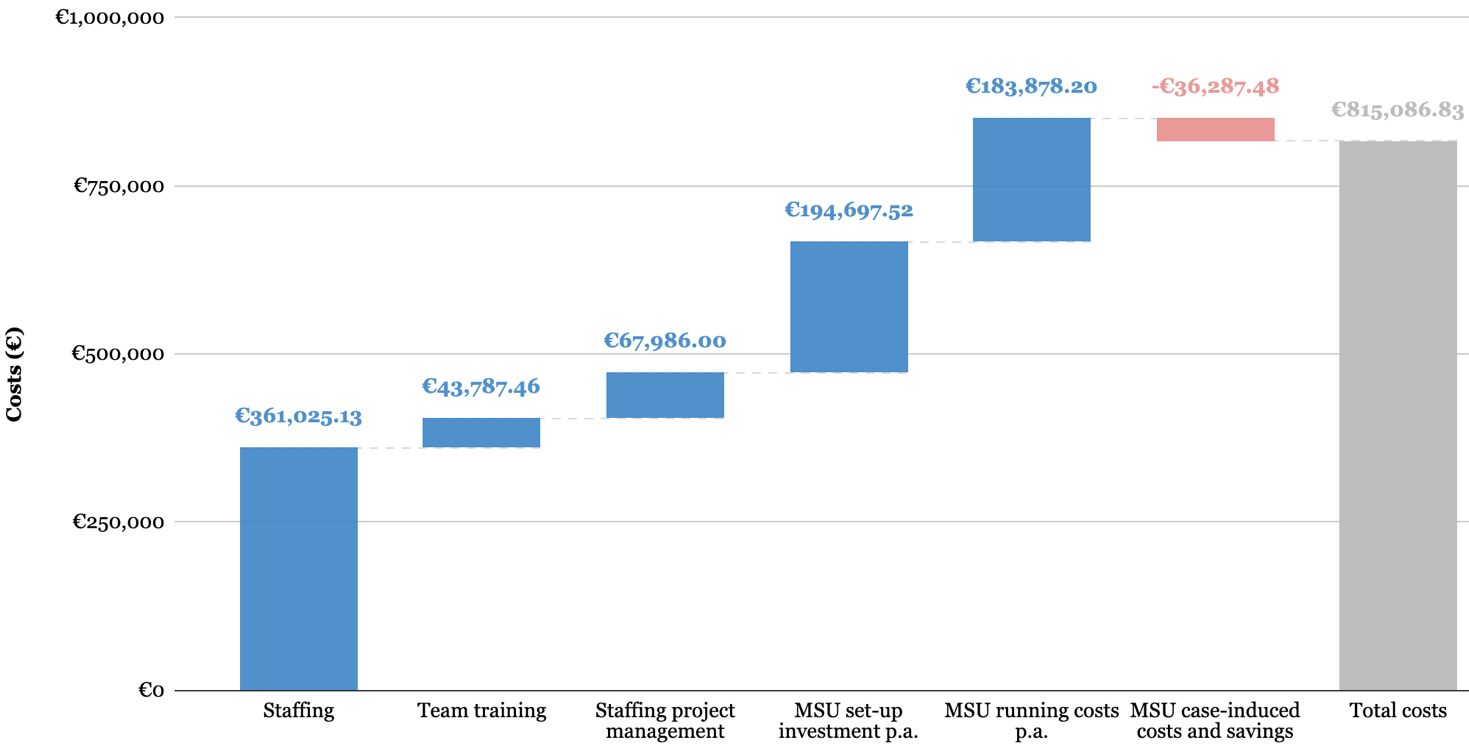
**

# Patient demographics

|  | **All patients with acute ischemic and hemorrhagic stroke** | **All patients with acute ischemic stroke** | **Subgroup: IVT treatment** |
| --- | --- | --- | --- |
| **Patient number** | N=6986 | N=5221 | N=1175 |
| **Age average / SD** | 70.3 (± 14.2) years | 71.9 (± 13.5) years | 72.2 (± 13.8) years |
| **% female** | 46.7% | 44.8% | 42.6% |

**Table S2:** Patient demographics

# Expected patient numbers per final diagnosis

|  | **Average number of ischemic stroke patients / day** | **Average IVT treatments / day** | **Average TIA / ICH / mimics patients** | **Average MSU costs per ischemic stroke patient / all patients** |
| --- | --- | --- | --- | --- |
| **8h operation** | 0.55 (0.44-0.67) | 0.12 (0.09-0.14) | 0.35 / 0.08 / 0.99 | € 7,809 / 3,109 |
| **10h operation** | 0.64 (0.51-0.76) | 0.13 (0.11-0.16) | 0.40 / 0.10 / 1.13 | € 7,907 / 3,148 |
| **16h operation** | 0.77 (0.61-0.92) | 0.16 (0.13-0.19) | 0.48 / 0.12 / 1.36 | € 7,739 / 3,081 |
| **24h operation** | 0.84 (0.68-1.01) | 0.18 (0.14-0.21) | 0.53 / 0.13 / 1.51 | € 9,273 / 3,692 |

**Table S3:** Display of the expected patient numbers daily, according to mode of operation. Also, average per-patient costs of MSU use were calculated for ischemic stroke patients and for all patients.

# Adherence to the STROBE Checklist

|  | | | **Item No** | **Recommendation** | **Page  No (in manuscript file)** | |  |
| --- | --- | --- | --- | --- | --- | --- | --- |
| **Title and abstract** | | | 1 | (*a*) Indicate the study’s design with a commonly used term in the title or the abstract | 1 and 2 | |  |
|  |  |  |  | (*b*) Provide in the abstract an informative and balanced summary of what was done and what was found | 2 | |  |
| **Introduction** | | | | | | |  |
| Background/rationale | | | 2 | Explain the scientific background and rationale for the investigation being reported | 3 | |  |
| Objectives | | | 3 | State specific objectives, including any prespecified hypotheses | 3 | |  |
| **Methods** | | | | | | |  |
| Study design | | | 4 | Present key elements of study design early in the paper | 4-5 | |  |
| Setting | | | 5 | Describe the setting, locations, and relevant dates, including periods of recruitment, exposure, follow-up, and data collection | 4 | |  |
| Participants | | | 6 | (*a*) *Cohort study*—Give the eligibility criteria, and the sources and methods of selection of participants. Describe methods of follow-up  *Case-control study*—Give the eligibility criteria, and the sources and methods of case ascertainment and control selection. Give the rationale for the choice of cases and controls  *Cross-sectional study*—Give the eligibility criteria, and the sources and methods of selection of participants | 4 | |  |
|  |  |  |  | (*b*) *Cohort study*—For matched studies, give matching criteria and number of exposed and unexposed  *Case-control study*—For matched studies, give matching criteria and the number of controls per case | N/A | |  |
| Variables | | | 7 | Clearly define all outcomes, exposures, predictors, potential confounders, and effect modifiers. Give diagnostic criteria, if applicable | 4 | |  |
| Data sources/ measurement | | | 8* | For each variable of interest, give sources of data and details of methods of assessment (measurement). Describe comparability of assessment methods if there is more than one group | 4-5; Table S1 | |  |
| Bias | | | 9 | Describe any efforts to address potential sources of bias | - | |  |
| Study size | | | 10 | Explain how the study size was arrived at | 4 | |  |
| Quantitative variables | | | 11 | Explain how quantitative variables were handled in the analyses. If applicable, describe which groupings were chosen and why | N/A | |  |
| Statistical methods | | | 12 | (*a*) Describe all statistical methods, including those used to control for confounding | 4, Figure legend 2 | |  |
|  |  |  |  | (*b*) Describe any methods used to examine subgroups and interactions | N/A | |  |
|  |  |  |  | (*c*) Explain how missing data were addressed | 4, Table S1 | |  |
|  |  |  |  | (*d*) *Cohort study*—If applicable, explain how loss to follow-up was addressed  *Case-control study*—If applicable, explain how matching of cases and controls was addressed  *Cross-sectional study*—If applicable, describe analytical methods taking account of sampling strategy | N/A | |  |
|  |  |  |  | (*e*) Describe any sensitivity analyses | N/A | |  |
| **Results** | | | | | | | |
| Participants | | 13* | (a) Report numbers of individuals at each stage of study—eg numbers potentially eligible, examined for eligibility, confirmed eligible, included in the study, completing follow-up, and analysed | | | | 6 |
|  |  |  | (b) Give reasons for non-participation at each stage | | | | N/A |
|  |  |  | (c) Consider use of a flow diagram | | | | N/A |
| Descriptive data | | 14* | (a) Give characteristics of study participants (eg demographic, clinical, social) and information on exposures and potential confounders | | | | Table S2 |
|  |  |  | (b) Indicate number of participants with missing data for each variable of interest | | | | 6 |
|  |  |  | (c) *Cohort study*—Summarise follow-up time (eg, average and total amount) | | | | N/A |
| Outcome data | | 15* | *Cohort study*—Report numbers of outcome events or summary measures over time | | | | N/A |
|  |  |  | *Case-control study—*Report numbers in each exposure category, or summary measures of exposure | | | | N/A |
|  |  |  | *Cross-sectional study—*Report numbers of outcome events or summary measures | | | | 6 |
| Main results | | 16 | (*a*) Give unadjusted estimates and, if applicable, confounder-adjusted estimates and their precision (eg, 95% confidence interval). Make clear which confounders were adjusted for and why they were included | | | | 6 |
|  |  |  | (*b*) Report category boundaries when continuous variables were categorized | | | | N/A |
|  |  |  | (*c*) If relevant, consider translating estimates of relative risk into absolute risk for a meaningful time period | | | | N/A |
| Other analyses | | 17 | Report other analyses done—eg analyses of subgroups and interactions, and sensitivity analyses | | | | 6 |
| **Discussion** | | | | | | | |
| Key results | | 18 | Summarise key results with reference to study objectives | | | | 7 |
| Limitations | | 19 | Discuss limitations of the study, taking into account sources of potential bias or imprecision. Discuss both direction and magnitude of any potential bias | | | | 8 |
| Interpretation | | 20 | Give a cautious overall interpretation of results considering objectives, limitations, multiplicity of analyses, results from similar studies, and other relevant evidence | | | | 8 |
| Generalisability | | 21 | Discuss the generalisability (external validity) of the study results | | | | 8 |
| **Other information** | | | | | | | |
| Funding | | 22 | Give the source of funding and the role of the funders for the present study and, if applicable, for the original study on which the present article is based | | | | 9 |

# Sources (supplementary material)

1. Quality report 2019, Diakonissenkrankenhaus Mannheim. Accessed April 5, 2023. https://www.diako-mannheim.de/diako/ueber-uns/qualitaetsmanagement/#tab_b4874ddfea49f9cc898b1a2b59395e63_4

2. Quality report 2019, University Medical Center Mannheim. Accessed March 30, 2023. https://www.umm.de/unternehmen/qualitaetsmanagement/qualitaetsbericht/

3. Mannheim - Einwohnerzahl bis 2021. Statista. Accessed March 27, 2023. https://de.statista.com/statistik/daten/studie/375304/umfrage/entwicklung-der-gesamtbevoelkerung-in-mannheim/

4. ORS-Karten. Accessed October 4, 2023. https://maps.openrouteservice.org/#/reach

5. Daten zu Schlaganfällen in der Stadt Erlangen 2021. Accessed December 27, 2022. https://www.gbe-bund.de/gbe/ergebnisse.prc_tab?fid=8299&suchstring=&query_id=&sprache=D&fund_typ=TAB&methode=&vt=&verwandte=1&page_ret=0&seite=1&p_lfd_nr=2&p_news=&p_sprachkz=D&p_uid=gast&p_aid=70345628&hlp_nr=2&p_janein=J

6. Fassbender K, Phillips DJ, Grunwald IQ, et al. Hybrid‐Mobile Stroke Unit: Opening the Indication Spectrum for Stroke Mimics and Beyond. *Stroke Vasc Interv Neurol*. 2023;3(1):e000482. doi:10.1161/SVIN.122.000482

7. Ellens NR, Schartz D, Rahmani R, et al. Mobile Stroke Unit Operational Metrics: Institutional Experience, Systematic Review and Meta-Analysis. *Front Neurol*. 2022;13. Accessed December 16, 2022. https://www.frontiersin.org/articles/10.3389/fneur.2022.868051

8. Mattila OS, Puolakka T, Ritvonen J, et al. Targets for improving dispatcher identification of acute stroke. *Int J Stroke Off J Int Stroke Soc*. 2019;14(4):409-416. doi:10.1177/1747493019830315

9. Mould-Millman NK, Meese H, Alattas I, et al. Accuracy of Prehospital Identification of Stroke in a Large Stroke Belt Municipality. *Prehosp Emerg Care*. 2018;22(6):734-742. doi:10.1080/10903127.2018.1447620

10. Nour M, Kazan C, Steeneken N, et al. Abstract 35: Dispatcher Impression of Stroke: Concordance With Paramedic Identification and Effect on Allocation of Standard Ambulances and Mobile Stroke Units. *Stroke*. 51(Suppl_1):A35-A35. doi:10.1161/str.51.suppl_1.35

11. Eliakundu AL, Cadilhac DA, Kim J, et al. Determining the sensitivity of emergency dispatcher and paramedic diagnosis of stroke: statewide registry linkage study. *J Am Coll Emerg Physicians Open*. 2022;3(4):e12750. doi:10.1002/emp2.12750

12. Bohm K, Kurland L. The accuracy of medical dispatch - a systematic review. *Scand J Trauma Resusc Emerg Med*. 2018;26(1):94. doi:10.1186/s13049-018-0528-8

13. Weinberg JH, Sweid A, DePrince M, et al. The impact of the implementation of a mobile stroke unit on a stroke cohort. *Clin Neurol Neurosurg*. 2020;198:106155. doi:10.1016/j.clineuro.2020.106155

14. Ebinger M, Siegerink B, Kunz A, et al. Association Between Dispatch of Mobile Stroke Units and Functional Outcomes Among Patients With Acute Ischemic Stroke in Berlin. *JAMA*. 2021;325(5):454-466. doi:10.1001/jama.2020.26345

15. Dietrich M, Walter S, Ragoschke-Schumm A, et al. Is prehospital treatment of acute stroke too expensive? An economic evaluation based on the first trial. *Cerebrovasc Dis Basel Switz*. 2014;38(6):457-463. doi:10.1159/000371427

16. Gyrd-Hansen D, Olsen KR, Bollweg K, Kronborg C, Ebinger M, Audebert HJ. Cost-effectiveness estimate of prehospital thrombolysis: results of the PHANTOM-S study. *Neurology*. 2015;84(11):1090-1097. doi:10.1212/WNL.0000000000001366

17. Online-Version des EBM. Published March 1, 2023. Accessed March 27, 2023. https://www.kbv.de/html/online-ebm.php

18. DFG-Programmpauschale - BMBF. Bundesministerium für Bildung und Forschung - BMBF. Accessed March 27, 2023. https://www.bmbf.de/bmbf/de/forschung/das-wissenschaftssystem/dfg-programmpauschale/dfg-programmpauschale_node.html

19. TV-Ärzte: § 8 Ausgleich für Sonderformen der Arbeit - Bürgerservice. Accessed March 27, 2023. https://www.gesetze-bayern.de/Content/Document/TV_Aerzte-8

20. Rettungssanitäter Präsenzkurs | NAW Berlin | Zentrum für Notfallmedizinische Aus- und Weiterbildung Berlin | Staatlich anerkannte Rettungsdienstschule. Accessed March 27, 2023. https://www.naw-berlin.de/kurse/rettungsfachpersonal/rettungssanitaeter-ausbildung-in-berlin
